# Anti-neuroinflammatory effects of GPR55 antagonists in LPS-activated primary microglial cells

**DOI:** 10.1186/s12974-018-1362-7

**Published:** 2018-11-19

**Authors:** Soraya Wilke Saliba, Hannah Jauch, Brahim Gargouri, Albrecht Keil, Thomas Hurrle, Nicole Volz, Florian Mohr, Mario van der Stelt, Stefan Bräse, Bernd L. Fiebich

**Affiliations:** 1 Neuroimmunology and Neurochemistry Research Group, Department of Psychiatry and Psychotherapy, Medical Center - University of Freiburg, Faculty of Medicine, University of Freiburg, Freiburg, Germany; 2 Institute of Organic Chemistry, Karlsruhe Institute of Technology (KIT), Karlsruhe, Germany; 3 Institute of Toxicology and Genetics, Karlsruhe Institute of Technology (KIT), Hermann-von-Helmholtz-Platz 1, 76344 Eggenstein-Leopoldshafen, Germany; 4 Department of Molecular Physiology, Leiden Institute of Chemistry, Leiden University, Leiden, the Netherlands; 5 Department of Psychiatry and Psychotherapy, Laboratory of Translational Psychiatry, University Hospital Freiburg, Hauptstr. 5, 79104 Freiburg, Germany

**Keywords:** GPR55, Prostaglandin E_2_, Cyclooxygenase, Microglia, Neuroinflammation

## Abstract

**Background:**

Neuroinflammation plays a vital role in Alzheimer’s disease and other neurodegenerative conditions. Microglia are the resident mononuclear immune cells of the central nervous system, and they play essential roles in the maintenance of homeostasis and responses to neuroinflammation. The orphan G-protein-coupled receptor 55 (GPR55) has been reported to modulate inflammation and is expressed in immune cells such as monocytes and microglia. However, its effects on neuroinflammation, mainly on the production of members of the arachidonic acid pathway in activated microglia, have not been elucidated in detail.

**Methods:**

In this present study, a series of coumarin derivatives, that exhibit GPR55 antagonism properties, were designed. The effects of these compounds on members of the arachidonic acid cascade were studied in lipopolysaccharide (LPS)-treated primary rat microglia using Western blot, qPCR, and ELISA.

**Results:**

We demonstrate here that the various compounds with GPR55 antagonistic activities significantly inhibited the release of PGE_2_ in primary microglia. The inhibition of LPS-induced PGE_2_ release by the most potent candidate KIT 17 was partially dependent on reduced protein synthesis of mPGES-1 and COX-2. KIT 17 did not affect any key enzyme involved on the endocannabinoid system. We furthermore show that microglia expressed GPR55 and that a synthetic antagonist of the GPR receptor (ML193) demonstrated the same effect of the KIT 17 on the inhibition of PGE_2_.

**Conclusions:**

Our results suggest that KIT 17 is acting as an inverse agonist on GPR55 independent of the endocannabinoid system. Targeting GPR55 might be a new therapeutic option to treat neurodegenerative diseases with a neuroinflammatory background such as Alzheimer’s disease, Parkinson, and multiple sclerosis (MS).

**Electronic supplementary material:**

The online version of this article (10.1186/s12974-018-1362-7) contains supplementary material, which is available to authorized users.

## Introduction

For many years, neuroinflammation has been known as a common phenomenon in the pathology of many brain diseases. Microglia, the principal cells involved in the innate immune response in the central nervous system (CNS), play essential roles in the maintenance of homeostasis and responses to inflammatory stimulus [1–3]. The over-activated microglia has been associated with neurodegenerative diseases such as Alzheimer’s disease (AD), Parkinson disease (PD), and traumatic brain injury or aging [4, 5].

The GPR55 is an orphan G-protein-coupled receptor, first described by Sawzdargo et al. in 1999 [6], and it is not only highly expressed in CNS, but also in peripheral tissue [7]. It can be activated by cannabinoids (CB) and non-CB, leading to the hypothesis that it might be a putative “type-3” cannabinoid receptor [8]. However, in contrast to the cannabinoid receptors CB1 and CB2, GPR55 only couples to Gα_12,13_ proteins which leads to the activation of the ras homolog gene family member A (RhoA) and Rho-associated protein kinase (ROCK). The phospholipase C pathway is triggered by these proteins, which increase the intercellular Ca^2+^ and extracellular signal-regulated kinase (ERK) phosphorylation [9].

GPR55 is expressed in immune cells, such as monocytes, natural killer (NK) cells [10], and microglia [11], and its involvement in inflammation has been reported [10, 12, 13]. Activation of GPR55 by the agonist O-1602 increased pro-inflammatory cytokines and cell cytotoxicity in monocytes and NK cells stimulated with LPS [10]. Corroborating with this, an antagonist of GPR55, CID16020046, and GPR55^−/−^ knockout mice decreased the pro-inflammatory cytokines in colitis mice models comparable to human inflammatory bowel disease (IBD) [12]. In hyperalgesia associated with inflammatory and neuropathic pain, it was observed to increase anti-inflammatory cytokines, IL-4 and IL-10, and also pro-inflammatory IFN-γ in GPR55^−/−^ knockout mice [13]. On the other hand, the GPR55 agonist O-1602 decreased IL-6 and TNF-*α* in a model of experimental acute pancreatitis [14].

The roles of GPR55 in the pathophysiology of the CNS are still not clear. In an excitotoxicity in vitro model of rat organotypic hippocampal slice cultures (OHSC), the activation of GPR55 receptor by L-α-Lysophosphatidylinositol (LPI) mediated neuroprotection through microglia [15]. In a mouse model of PD, the expression of GPR55 was downregulated in the striatum and the treatment with an agonist of GPR55, abnormal-cannabidiol, improved the motor behavior by neuroprotection of dopaminergic neuron cell bodies [16]. A molecular, anatomical, electrophysiological, and behavioral study of GPR55^−/−^ knockout mice demonstrated a normal development of brain structure and did not affect the endocannabinoid system nor muscle strength and motor learning. However, these mice presented deficits in motor coordination and thermal sensitivity [17].

These studies suggest that GPR55 signaling can be involved in neurodegeneration and modulate certain cytokines and thus inflammation. However, its effects on neuroinflammation, especially on the production of members of the arachidonic acid pathway in activated microglia, have not been elucidated in detail. We therefore studied the effects of novel synthesized GPR55 antagonists in LPS-activated microglia by examining prostaglandin E_2_ (PGE_2_) production, COX/mPGES-1 mRNA, and protein levels. Furthermore, we evaluated the effect of KIT17 on key enzymes involved in the endocannabinoid system such as diacylglycerol lipase-α(DAGLα), monoacylglycerol lipase (MAGL), α,β-hydrolase domain-containing 6 and 12 (ABHD6 and ABHD12), and fatty acid amide hydrolase (FAAH).

## Methods

### Ethics statement

Animals were obtained from the Center for Experimental Models and Transgenic Services-Freiburg (CEMT-FR). All the experiments were approved and conducted according to the guidelines of the ethics committee of the University of Freiburg Medical School under protocol no. X-13/06A, and the study was carefully planned to minimize the number of animals used and their suffering. For the activity-based protein profiling (ABPP), the experiments were performed at Leiden University according to guidelines approved by the ethical committee of Leiden University (DEC#13191).

### Chemicals

**Fig. 1:**
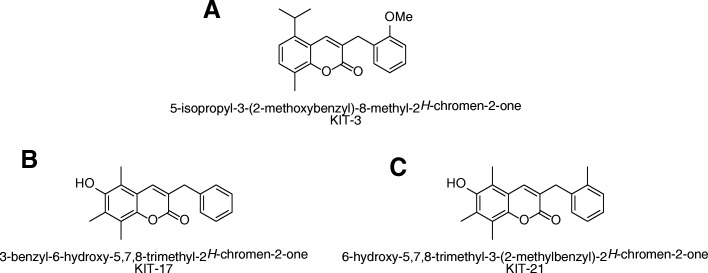
Molecular structure of the synthesized compounds **a** KIT 3: 8-isopropyl-3-(2-methoxybenzyl)-5-methyl-2*H*-chromen-2-one, MW = 336,1725 g/mol. **b** KIT 17: 3-benzyl-6-hydroxy-7,8-dimethyl-2*H*-chromen-2-one, MW = 294,1256 g/mol. **c** KIT 21: 6-hydroxy-7,8-dimethyl-3-(2-methylbenzyl)-2*H*-chromen-2-one, MW = 308,1412 g/mol

Synthetic GPR55 antagonists (Fig. 1) were synthesized at the Institute for Organic Chemistry Karlsruhe - KIT (Karlsruhe, Germany) [18] and dissolved in DMSO. The number of the corresponding compounds in the Rempel et al. 2013 paper are: KIT3 is 14, KIT17 is 37, and KIT21 is 41. ML193 and O-1602 were obtained from Tocris Biosciences. LPS from *Salmonella typhimurium* (Sigma Aldrich, Deissenhofen, Germany) was resuspended in sterile phosphate-buffered saline (PBS, 5 mg/mL) as stock and subsequently used at a final concentration (10 ng/mL) in the cultures.

### Primary microglia cultures

As described in our previous studies [19–24], primary mixed glial cell cultures were prepared from cerebral cortices of 1-day neonatal Sprague-Dawley rats. Under sterile conditions, the brains were carefully taken, and the cerebral cortices were isolated and the meninges removed. Then, the cortices were gently dissociated and filtered through a 70-μm nylon cell strainer (BD biosciences, Heidelberg, Germany). After centrifugation at 1000 rpm for 10 min, cells were collected and resuspended in Dulbecco’s modified Eagle’s medium (DMEM) containing 10% fetal calf serum (Biochrom AG, Berlin, Germany) and antibiotics (40 U/mL penicillin and 40 μg/mL streptomycin, both from PAA Laboratories, Linz, Austria). Cells were cultured on 10-cm cell culture dishes (Falcon, Heidelberg, Germany) with density of 5 × 10^5^ cells/mL in 10% CO_2_ at 37 °C (Heracell 240i, Thermo Scientific). After 12 days in vitro, floating microglia were harvested and re-seeded into 7  cm^2^ culture flask to give pure microglial cultures. On the next day, medium was changed to remove non-adherent cells, and after 1 h, the cells were stimulated for respective experiments.

### Determination of PGE_2_ release from LPS-activated microglia

Cultured primary rat microglia were incubated with synthesized compounds (KITs) (0.1–25 μM) or commercial antagonist ML193 (10 μM) or agonist O-1602 (0.1–10 μM) for 30 min. Afterwards, the cells were treated with or without LPS (10 ng/mL) for the next 24 h. Supernatants were harvested and levels of PGE_2_ were measured using a commercially available enzyme immunoassay (EIA) kit (Assay Designs Inc., Ann Arbor, MI, USA; distributed by Biotrend, Cologne, Germany). The results were normalized to LPS and presented as percentage of change in PGE_2_ levels of at least three independent experiments.

### Cell viability assay

Viability of primary rat microglia after treatment with the synthesized compounds was measured by the CellTiter-Glo® Luminescent Cell Viability Assay (Promega), which is used to determine the number of metabolically active and viable cells in cell culture based on quantitation of the ATP present in the cells. Briefly, cells were cultured in 96 well plates at the density of 25 × 10^3^ cells/well for 24 h. Then, the medium was changed and after at least 1 h, the cells were incubated with KIT 3, KIT 17, and KIT 21 for 24 h. The compounds were dissolved in DMSO, and DMSO was used in the negative control wells at final concentration of 0.15% and as a positive control in a higher concentration (10%) during experiments. The concentration of ATP was measured after 24 h of incubation by adding 100 μL of reconstituted substrate and incubating for 10 min. Luminescence was measured using a Modulus™ II Microplate Multimode Reader (Turner BioSystems, USA).

### RNA isolation and quantitative PCR

Quantitative real-time PCR (qPCR) was performed to determine the presence of *GPR55* in microglia and transcriptional regulation of *COX-1*, *COX-2*, and *mPGES-1* by synthesized compound (KIT17) in activated microglia. Cultured primary rat microglia were left untreated or incubated with LPS (10 ng/mL) in the presence or absence of KIT 17 (0.1–10 μM) which was added 30 min before LPS (10 ng/mL) treatment for 4 h. Total RNA was then extracted using the guanidine isothiocyanate method [25]. The cDNA synthesis were reverse transcribed from 1 μg of total RNA using Moloney Murine Leukemia Virus (M-MLV) reverse transcriptase (Promega, Mannheim, Germany), RNase Inhibitor rRNasin® (Promega), dNTP master mix (Invitek, Berlin, Germany), and random hexamer primers (Promega). The real-time PCR amplification was carried out by the CFX96 real-time PCR detection system (Bio-Rad Laboratories, Inc.) using iQ™ SYBR™ Green supermix (Bio-Rad Laboratories GmbH, Munich, Germany). Reaction conditions were 3 min at 95 °C, followed by 40 cycles of 15 s at 95 °C, 30 s at 50 °C, and 45 s at 72 °C, and every cycle was followed by plate reading. After that, 1 min at 95 °C, 1 min at 55 °C, followed by melt curve conditions of 65 °C, 95 °C with increment of 0.5 °C for 5 s, followed by final plate reading. Glyceraldehyde 3-phosphate dehydrogenase (GAPDH) served as an internal control for sample normalization, and the comparative cycle threshold Ct method was used for data quantification [26]. The primer sequences were as follows:

*GPR55:* Fwd 5´-ACGTGGAGTGCGAGAGTCTT-3′;

Rev 5′-TGCCCATAGGAAGGAGGAA-5′;

*COX-1:* Fwd 5’-GCTCTTCAAGGATGGGAAACT-3′;

Rev 5’-TTCTACGGAAGGTGGGTACAA-3′;

*COX-2:* Fwd 5′-GGCTTACAAGACGCCACATCACCT-3′;

Rev 5′-TGGTTTAGGCGGCCGGGGAT-3′;

*mPGES-1:* Fwd 5′-TGCAGCACGCTGCTGGTCAT-3′;

Rev 5′-GTCGTTGCGGTGGGCTCTGAG-3′;

*GAPDH:* Fwd 5′-ATGCTGGTGCTGAGTATGTC-3′;

Rev 5′-AGTTGTCATATTTCTCGTGGGTT-3′.

### Immunoblotting

Rat primary microglia were treated with KIT 17 (0.1–10 μM) and control for 30 min; then, the LPS (10 ng/mL) was added for 24 h. After the experiment, the cells were washed with cold PBS and lysed in the lysis buffer (42 mM Tris–HCl, 1.3% sodium dodecyl sulfate, 6.5% glycerin, 100-μM sodium orthovanadate, and 2% phosphatase and protease inhibitors). Protein concentration of the samples was measured using the bicinchoninic acid (BCA) protein assay kit (Thermo Fisher Scientific, Bonn, Germany) according to the manufacturer’s instructions. For Western blotting, 10–20 μg of total protein from each sample was subjected to sodium dodecyl sulfate-polyacrylamide gel electrophoresis (SDS-PAGE) under reducing conditions. Afterward, proteins were transferred onto polyvinylidene fluoride (PVDF) membranes (Merck Millipore, Darmstadt, Germany) by semi-dry blotting. After blocking with Roti-Block (Roth, Karlsruhe, Germany), membranes were incubated overnight with primary antibodies. Primary antibodies were goat anti-COX-2 (1:500; Santa Cruz Biotechnology, Heidelberg, Germany), rabbit anti-mPGES-1 (1:6000; Agrisera, Vännas, Sweden), and rabbit anti-actin (1:5000; Sigma Aldrich). The proteins were detected with horseradish peroxidase-coupled rabbit anti-goat IgG (Santa Cruz, 1:100,000 dilution) or goat anti-rabbit IgG (Amersham, 1:25,000 dilution) using enhanced chemiluminescence (ECL) reagents (GE Healthcare, Freiburg, Germany). Densitometric analysis was performed using ImageJ software (NIH, USA), and β-actin control was used to confirm equal sample loading and normalization of the data.

### Cyclooxygenase activity assay in primary microglia culture

Under unstimulated conditions, primary microglial cells only express the COX-1 isoform [27]. To measure COX-1 activity, primary rat microglial cells were plated in 24-well cell culture plates. After 24 h, medium was removed and replaced with serum-free medium. KIT 17 (0.1–10 μM) or the selective reversible COX-1 inhibitor SC560 (1 and 10 μM) was added and left for 15 min. Then, 15 μM of arachidonic acid were supplemented for another 15 min. Supernatants were then collected and used for the determination of PGE_2_.

To measure COX-2 activity, primary rat microglial cells were plated in 24-well cell culture plates and pre-incubated with LPS (10 ng/mL) for 24 h. Then, medium was removed and replaced with serum-free medium. KIT 17 (0.1–10 μM) or diclofenac sodium (preferential COX-2 inhibitor, 10 μM) was added and left for 15 min. Then, 15 μM of arachidonic acid was supplemented for another 15 min. Supernatants were then collected and used for determination of PGE_2_.

### Activity-based protein profiling (ABPP) in mouse brain proteome

The mouse brain proteome preparation and gel-based ABPP was performed as previously described [28, 29]. Mouse tissues were homogenized in lysis buffer A (20 mM HEPES pH 7.2, 2 mM DTT, 1 mM MgCl_2_, 25 U/mL Benzonase) and incubated for 5 min on ice. To remove containing debris, the suspension was low speed centrifuged (× 2500*g*, 3 min, 4 °C). The resulting supernatant was subjected to ultracentrifugation (× 100.000*g*, 45 min. 4 °C, Beckman Coulter, Type Ti70 rotor) to separate the membrane fraction as a pellet and the cytosolic fraction in the supernatant. The pellet was resuspended in storage buffer (20 mM HEPES pH 7.2, 2 mM DTT). The total protein concentration was determined with Quick Start Bradford assay (Bio-Rad). Membranes and supernatant were stored in small aliquots at − 80 °C until use. For the gel-based ABPP, mouse brain membrane proteome (2 mg/mL) were pre-incubated with 0.5 μL vehicle (DMSO) or 0.5 μL KIT 17 (in DMSO) for 30 min at room temperature. Followed by treatment with the activity-based probes tetramethylrodamine 5-carboxamdio fluorophosphonate (TAMRA-FP, 100 nM final concentration) and MB064 (250 nM final concentration) for 15 min at room temperature. The reactions were quenched by addition of 10 μL 3 × SDS-PAGE sample buffer, and the samples were directly loaded and resolved on SDS-PAGE gel (10% acrylamide). The gels were scanned with a Bio-Rad Chemidoc (Bio-Rad Laboratories B.V.) using settings for Cy3 and TAMRA (excitation wavelength 532 nm, emission wavelength 580 nm) and analyzed with ImageLab (5.2.1, Bio-Rad Laboratories B.V.).

### Statistical analysis

Statistical analyses were performed using Prism 5 software (GraphPad software Inc., San Diego, CA, USA). Values of all experiments were represented as mean ± SEM of at least three independent experiments. Raw values were converted to percentage and compared using one-way ANOVA with post hoc Student-Newman-Keuls test (multiple comparisons). The level of significance was consider as *p < 0.05, **p < 0.01, and ***p < 0.001.

## Results

### Expression of GPR55 in primary rat microglia

To prove that microglial cells express *GPR55*, we studied the mRNA levels of *GPR55* in primary rat microglia with or without LPS stimulation. We observed the *GPR55* mRNA is expressed in microglial cells and the stimulation with 10 ng/mL LPS did not affect the expression of this receptor (Additional file 1).

### Effects of antagonists of the GPR55 receptor on PGE_2_ release in LPS-stimulated primary microglial cells

**Fig. 2:**
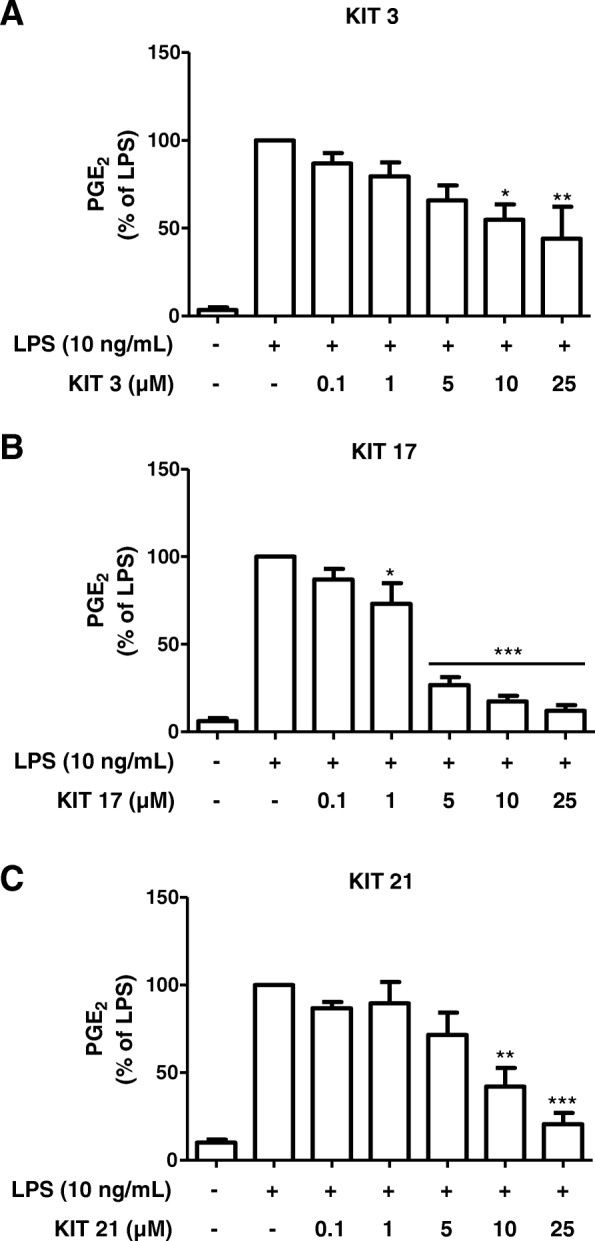
Effects of different KIT compounds on the release of PGE_2_ in LPS-activated microglia. Microglia were pre-treated with KIT 3 (**a**), KIT 17 (**b**), or KIT 21 (**c**) (0.1–25 μM) for 30 min; afterwards, cells were incubated with or without LPS (10 ng/mL) for the next 24 h. Cell supernatants were then collected, and release of PGE_2_ was measured by enzyme immune assay (EIA). Values are presented as the mean ± SEM of at least 3 independent experiments. Statistical analyses were carried out by using one-way ANOVA and Newman-Keuls post hoc test with *p < 0.01, **p < 0.05, and ***p < 0.001 compared to LPS group

We further investigated the effects of 21 chemical-related GPR55 receptor antagonists (KIT 1-KIT 21) on LPS-induced PGE_2_ synthesis in microglia cells. We selected some of the active KIT compounds, KIT 3, KIT 17, and KIT 21 (Fig. 1), from the first screening (Additional file 2) due to activity of 90–100% PGE_2_ inhibition and the availability of the compound for further studies. When treated with LPS, primary microglial cells produced robust amounts of PGE_2_ (2596.13 pg/mL, considered as 100%) compared to the untreated control (326.59 pg/mL) and this increase was inhibited by the three synthesized compounds (KIT 3, KIT 17, and KIT 21) in a concentration-dependent manner (Fig. 2).

All three compounds tested, statistically, prevented the increase on levels of PGE_2_ at the concentrations of 10 and 25 μM. Furthermore, the KIT 17 (Fig. 2b) also statistically prevented at the concentration of 1 and 5 μM.

### Effects of GPR55 receptor antagonists on cell viability

**Fig. 3:**
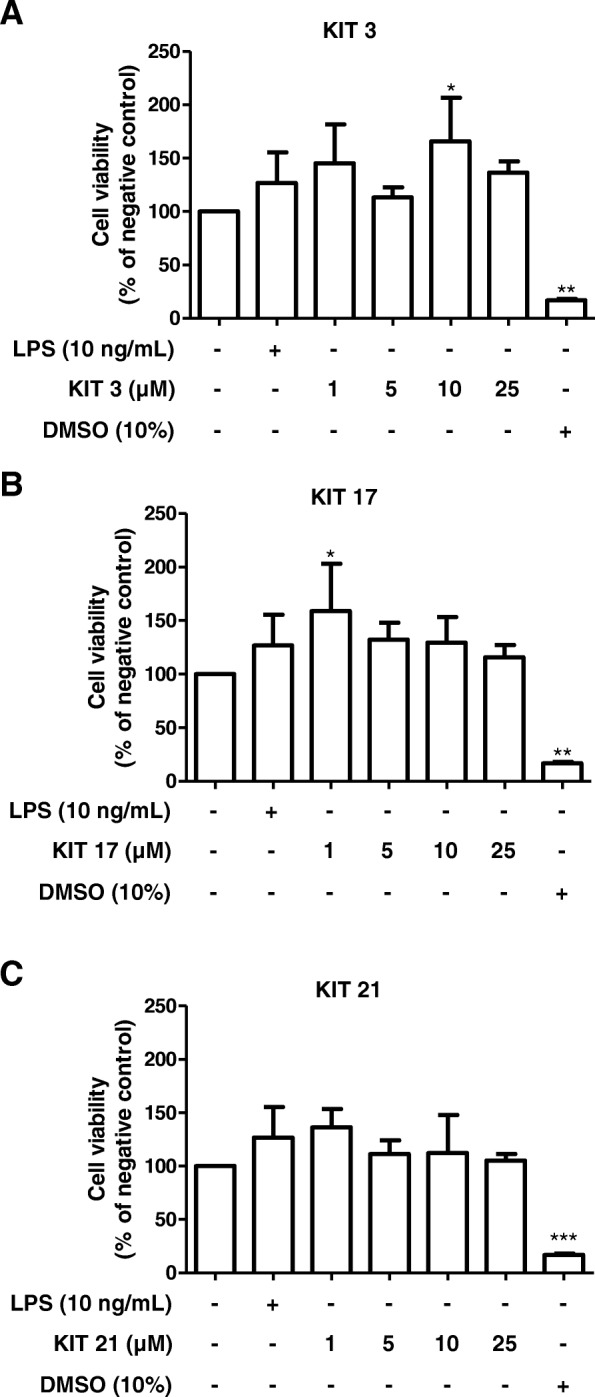
Evaluation of cell viability of microglial cells treated with different KIT compounds. Microglia cells were exposed with different concentrations of KIT 3 (**a**), KIT 17 (**b**), or KIT 21 (**c**) for the next 24 h. Cell viability was measured based on quantitation of ATP present in the cells, and data were normalized as percentage of negative control. Values are presented as the mean ± SEM of at least three independent experiments. Statistical analyses were carried out by using one-way ANOVA and Newman-Keuls post hoc test with *p < 0.05, **p < 0.01, and ***p < 0.001 compared to negative group

We performed a cell viability assay to exclude the possibility that the inhibitory effects of the synthesized compounds observed were due to a reduction of cell viability. The effects of KIT 3, KIT 17, and KIT 21 on cell viability were studied using an ATP assay in primary microglial cells. The incubation with LPS or the compounds did not change cell viability compared to vehicle (DMSO) (Fig. 3). A significant increase in ATP was observed with the concentration of 10 μM KIT 3 (Fig. 3a) and 1 μM KIT 17 (Fig. 3b). DMSO in the high concentration of 10% was used as positive control, and it strongly affected cell viability of microglial cells (Fig. 3, right column).

Since KIT 17 showed a desirable inhibitory profile on PGE_2_ release and had no negative impact on cell viability, even increased ATP levels, we used KIT 17 for the mechanistic follow-up experiments.

### KIT 17 as well as the known GPR55 antagonist ML potently inhibited LPS-induced PGE_2_ release whereas the GPR55 agonist O-1602 did not affect the PGE_2_ levels in microglial cells

**Fig. 4:**
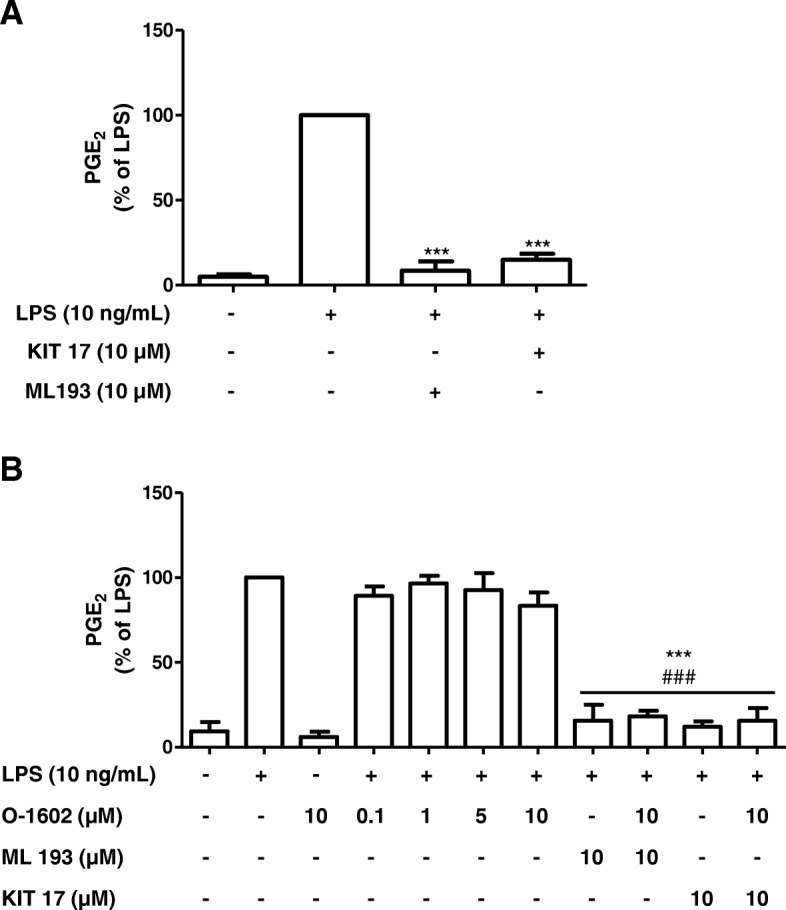
Effects of the commercial GPR55 antagonist ML193 (**a**) and agonist O-1602 alone and in combination with ML 193 and KIT17 (**b**) on PGE2 release in LPS-stimulated primary microglial cells compared to KIT 17 alone

We further investigate the effects of a commercial antagonist (ML193) and an agonist of GPR55 (O-1602) on PGE_2_ release in LPS-stimulated primary microglial cells. ML193 (10 μM) potently and statistically prevented LPS-induced increase of PGE_2_ levels by 90%, and a comparable inhibition was observed using KIT 17 (85% inhibition) (Fig. 4a). A potent agonist of GPR55, O-1602 (0.1–10 μM), did not affect the LPS-induced PGE_2_ release as well as did not interfere with the effects of ML193 and KIT 17. O-1602 also did not alter basal PGE_2_ levels (Fig. 4b).

### Activity-based protein profiling on mouse brain proteome for selectivity and off-target activity of KIT 17

**Fig. 5:**
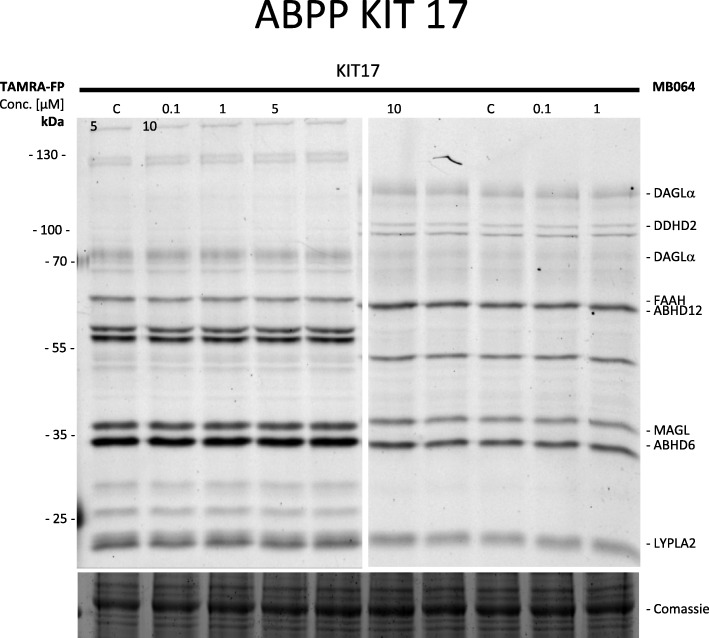
Effects of KIT 17 on key enzymes involved in the endocannabinoid system. Dose-response gel-based ABPP on mouse membrane brain proteome with activity-based probes TAMRA-FP (left) and MB064 (right) (*N* = 3). ABHD6 and ABHD12 α,β-hydrolase domain-containing 6 and 12; DAGLα diacylglycerol lipase-α; FAAH fatty acid amide hydrolase, and MAGL monoacylglycerol lipase

Our previous study demonstrated the effects of KITs compounds on GPR55, CB1, CB2, and GPR18 receptors [18], in which KIT 17 showed inhibitory effects on GPR55 and CB2 receptors. To investigate its involvement on other members the endocannabinoid system, we further evaluated the selectivity of KIT 17 on key enzymes involved in the endocannabinoid system [diacylglycerol lipase-αDAGLα, monoacylglycerol lipase (MAGL), α,β-hydrolase domain-containing 6 and 12 (ABHD6 and ABHD12), and fatty acid amide hydrolase (FAAH)]. To this end, we used the approach of gel-based activity-based protein profiling (ABPP) on a mouse membrane brain proteome. As shown in Fig. 5, we did not find any off-target interactions of KIT 17 in the concentrations of 0.1–10 μM. This indicates that KIT 17 showed a highly selective profile against GPR55 and CB2, as no significant reduction of any other endocannabinoid enzyme in the mouse brain proteome was observed in this experimental setting.

### Effects of KIT 17 on COX-2 and mPGES-1 mRNA and protein synthesis in LPS-stimulated primary microglial cells

**Fig. 6:**
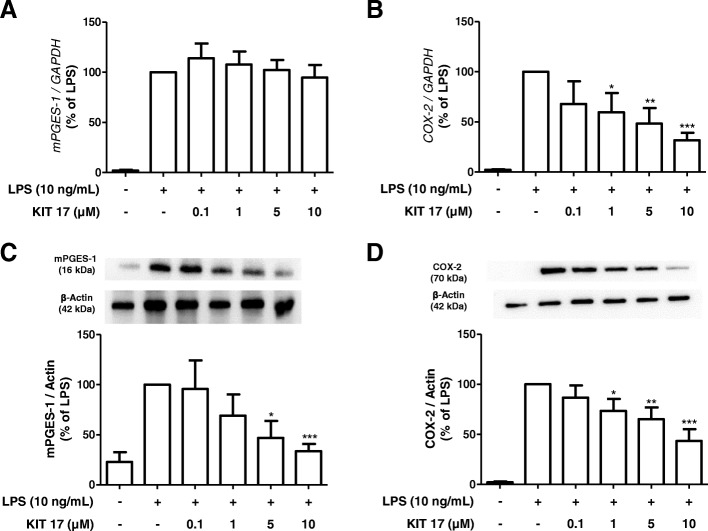
Effects of KIT 17 on mRNA expression and protein synthesis of mPGES-1 (**a**, **c**) and COX-2 (**b*,*** **d**) in LPS-stimulated primary microglial cells. **a**, **b** Cells were pre-treated with different concentrations of KIT 17 for 30 min before stimulating with LPS. After 4 h, *mPGES-1* (**a**) and *COX-2* (**b**) were measured by qPCR. Western blot analysis of protein levels of mPGES-1 (**c**) or COX-2 (**d**) in cells pre-treated with different concentrations of KIT 17 for 30 min following LPS stimulation for 24 h. Protein levels were normalized to β-actin loading control (*n* = 3–4). *p < 0.05, **p < 0.01, and ***p < 0.001 with respect to LPS (one-way ANOVA followed by the Newman-Keuls post hoc test)

Prostaglandins are synthesized through enzymes COX-2 and mPGES-1 under an inflammatory stimulus [30]. As shown in Fig. 6, LPS induced the expression and the protein synthesis of COX-2 and mPGES-1. We observed that LPS-induced mPGES-1 synthesis was significantly inhibited by KIT 17 in a concentration-dependent manner starting at the concentration of 5 μM and revealing maximal effects using 10 μM, which decreased mPGES-1 levels about approx. 70%, if compared to LPS control (Fig. 6c). However, no effects were observed on mRNA expression of *mPGES-1* at the 4-h time point (Fig. 6a).

Furthermore, we investigated whether KIT 17 affected COX-2 mRNA expression and protein synthesis. The pre-treatment with KIT 17 in microglial cells showed a significant concentration-dependent decrease of LPS-induced COX-2 mRNA expression (Fig. 6b) and protein synthesis (Fig. 6d) starting at 1 μM and maximal effects using 10 μM, which decreased approximately 60% and 30% of COX-2 mRNA and 70% and 40% of protein levels, respectively, if compared to the LPS control.

We also evaluated the effect of KIT 17 on *COX-1* mRNA expression (Additional file 3) showing a downregulation of *COX-1* mRNA expression by LPS stimulation and no modulatory effect of KIT17.

### Effects of KIT 17 on enzyme activity of COX-1 and COX-2

**Fig. 7:**
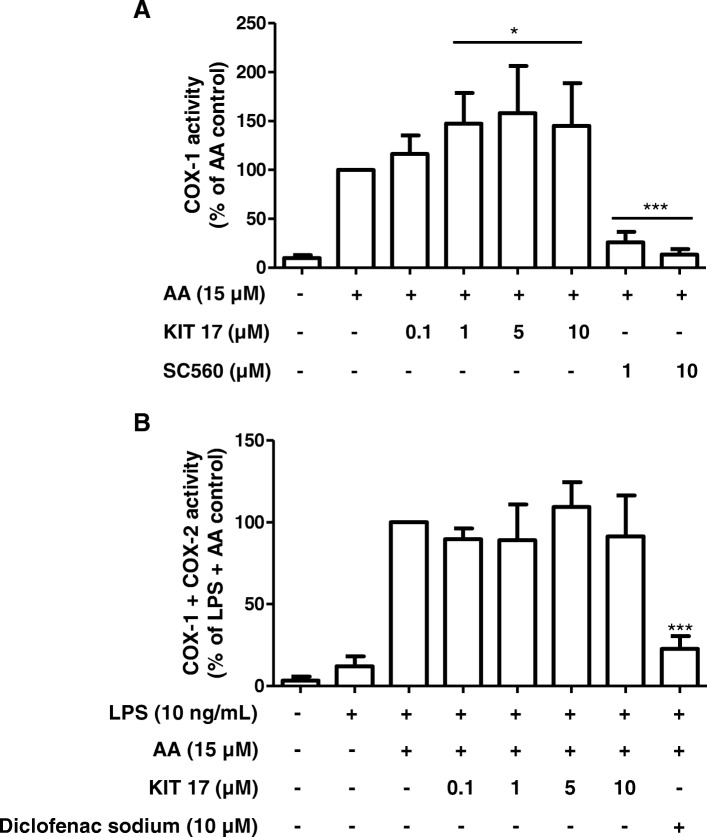
Effects of KIT 17 on COX-1 (A) and COX-2 (**b**) enzyme activity in primary microglia cells. **a** For COX-1 activity assay, cells were treated with different concentrations of KIT 17 for 15 min before addition of 15 μM of arachidonic acid. PGE_2_ in the supernatants was measured after additional 15 min. **b** Cells were stimulated for 24 h with LPS (10 ng/mL) and then treated with different concentrations of KIT 17 for 15 min. Then, 15 μM of arachidonic acid was added and PGE_2_ in the supernatants was measured by enzyme immune assay (EIA). Data are expressed as mean ± S.E.M. of at least 3 independent experiments. *p < 0.05, **p < 0.01, and ***p < 0.001 with respect to control (one-way ANOVA followed by the Newman-Keuls post hoc test)

Thus, we extended our study to investigate if these robust inhibitory effects of KIT 17 on PGE_2_ release were additionally due to a direct suppression of COX enzymatic activity. As shown in Fig. 7a, COX-1 activity was even increased by KIT 17 in the concentrations of 0.1–10 μM, whereas in COX-2 activity was not affected (Fig. 7b). The control inhibitors for COX-1 (SC560) and COX-2 (diclofenac) showed the expected inhibitory effects (last 2 columns on the right).

## Discussion

Neuroinflammatory processes are considered a double-edged sword, having both protective and detrimental effects in the brain [31–33]. Microglia, the resident innate immune cells of the brain, are a key component of neuroinflammatory response. There is a growing interest in developing drugs to target microglia and control neuroinflammatory processes [34, 35]. We have developed a new series of GPR55 receptor antagonist compounds derived from the coumarin structure which, in general, exhibit a promising profile as GPR55 receptor ligands with antagonistic activity [18].

In the current study, we demonstrated the anti-neuroinflammatory effect of these synthesized compounds in LPS-activated primary microglial cells by inhibiting PGE_2_ release and downregulating mPGES-1 and COX-2 protein levels. We furthermore show that microglia expressed *GPR55* and that a synthetic antagonist of the GPR55 receptor (ML193) revealed comparable effects as KIT 17 on the inhibition of LPS-induced PGE_2_. Furthermore, we demonstrated that KIT 17 did not affect any enzyme involved in the endocannabinoid system in the mouse brain proteome.

Monocytes and macrophages express an extensive repertoire of G-protein-coupled receptors (GPCRs) that regulate inflammation and immunity [11, 36]. Among these receptors, the role of GPR55 in the perspective of neurological diseases and in microglia activation is controversial. GPR55 is expressed in many mammalian tissues including several brain regions [6, 37]. Pietr et al. (2009) showed that *GPR55* mRNA is significantly expressed in both, primary mouse microglia and the BV-2 mouse microglial cell line, and the stimulation with LPS downregulated *GPR55* expression [11]. However, in a study using primary rat microglial cells culture, the GPR55 transcript was detected in non-stimulated and LPS-stimulated cells, demonstrating no alterations of its levels [38]. Collaborating with this finding, we were able to confirm the expression of GPR55 in primary rat microglia and that the stimulation with LPS did not affect the expression of the receptor. Further studies, probably using microglia cell lines, have to be performed to demonstrate the functional GPR55 receptors on microglial cells**.**

We next studied the potential role of GPR55 in microglia-mediated neuroinflammation by studying the effects of the KIT compounds on prostaglandin synthesis. Microglial cells are the resident macrophages of the CNS and the most important source of PGE_2_ in neuroinflammation. As shown before, the stimulation of primary microglia with LPS strongly increases PGE_2_ and COX-2 expression [39–41].

The main finding of our study is that the various synthesized KIT compounds decreased PGE_2_ release in LPS-treated microglia and upstream members of the arachidonic acid pathway (COX-2/mPGES-1) without affecting the viability of microglia. Moreover, the similar inhibition of PGE_2_ was observed using the commercially available GPR55 antagonist ML193. Interestingly, the agonist O-1602 did not affect the inhibitory effect of ML193 as well as KIT 17 and also showed no effect on basal PGE_2_ levels when applied without LPS.

KIT 17 and the other KIT compounds tested were synthesized by Rempel and collaborators (2013) as a series of coumarin derivatives (KITs) targeting GPR55 (formerly also known as CB3 receptor).

However, the compounds, including KIT 17, also bind to other receptors such as CB1, CB2, and GPR18 receptors, suggesting that the observed activities of KIT 17 are possible mediated also by other receptors [18]. The inhibitory values for KIT 17 on GPR55 is 9.32 ± 1.05 [IC50 (μM ± SEM)] but it also binds to CB2 [Ki ± SEM (μM) 3.42 ± 0.90], whereas only weak binding was observed for CB1 and GPR18 [Ki ± SEM (μM)/IC50 ≥ 10] [18]. These data suggest that we can exclude CB1 and GPR18 as potential other receptors involved in the anti-inflammatory effects observed in microglial cells, but we cannot exclude an additional or exclusive involvement of CB2. The fact that KIT 17 as a GPR55 or CB2 antagonist is inhibiting TLR4-mediated inflammation, suggests an inverse agonistic activity of KIT 17 on GPR55 or CB2. To confirm this hypothesis and to prove a possible involvement of CB2, we used AM630, a compound with CB2 inverse agonistic activity with a Ki of 32 nM [42], also showing antagonistic activities on CB2. As shown in Additional file 4, AM630 also inhibited LPS-induced PGE_2_ release in a comparable inhibitory profile on LPS-induced PGE_2_ as KIT 17 with a slight more activity using 1 μM. However, the Ki of AM630 (32 nM) is 100 fold lower towards CB2 then the one of KIT 17 (3.42 μM on CB2), implicating a more potent effect of AM630 on PGE_2_ release induced by LPS then KIT 17, if we assume a CB2-mediated effect. Since the inhibitory activity of AM630 on LPS-induced PGE_2_ release is only marginally higher than the effect of KIT 17, we conclude that CB2 is most likely not mediating the effects of KIT 17 and that AM630 might even also act via GPR55 on the observed effects. More studies have to be performed to support this conclusion, since there are to our knowledge no data available on the effects of AM630 on GPR55.

Since the GPR55 is described to be linked to the endocannabinoid system and due to the fact that the KIT compounds are also binding to CB1 and CB2, we were further interested in other interactions between KIT 17 and other members of the endocannabinoid system. Using the activity-based protein profiling on mouse brain proteome for selectivity and off-target activity approach, we did not find any interactions between KIT 17 and the endocannabinoid enzymes DAGLα, MAGL, ABHD6, ABHD12, and FAAH. An interaction or effects of GPR55 activation on these enzymes have not been described to our knowledge in the literature yet.

Thus, our data strongly suggest that PGE_2_ inhibitory effects in are most likely mediated by GPR55, although other mechanism independent of GPR55 and CBs in the observed effects cannot be excluded*.* The involvement of GPR55 in the activation of the arachidonic acid cascade during microglia activation and neuroinflammation has not been reported yet. The potential role in peripheral inflammation has been studied with GPR55 antagonists or knockout mice. The GPR55 inhibitor CID16020046 reduced TNF-*α*, IL-1*β*, IL-6, and COX-2 levels in a mouse model of intestinal inflammation [12]. In a mouse model of colorectal cancer, the levels of COX-2 and PGF_2α_ were decreased in GPR55^−/−^ [43]. In hyperalgesia associated with inflammatory and neuropathic pain, an increase of anti-inflammatory cytokines, IL-4, and IL-10 has been observed in GPR55^−/−^ knockout mice [13]. These data also suggest that GPR55 might be the mediator of the anti-neuroinflammatory effects of KIT17 as observed in this study.

In accordance with the diverse and complex pharmacology of GPR55, no data yet existed regarding the efficacy of GPR55 antagonists on COX-1/2 activity. Thus, we elucidated whether the robust inhibitory effects of KIT 17 on LPS-induced PGE_2_ release is due to a direct inhibition of COX enzymatic activity, the mechanism of action of most NSAIDs. We demonstrate here, that COX-1 activity was increased by KIT 17 in the doses of 1–25 μM, whereas in COX-2 activity was not affected. The control inhibitors for COX-1 (SC560) and COX-2 (diclofenac) showed the expected inhibitory effects. This suggests that the PGE_2_ inhibiting effects of KIT 17 and most likely by the other KIT compounds is not due a direct inhibition of COX activity. The COX-1 inducing effects might even have positive benefits, since COX-1 is protecting the gut mucosa and its inhibition by some NSAIDs cause gastro-intestinal side effects [44, 45].

## Conclusions

**Fig. 8:**
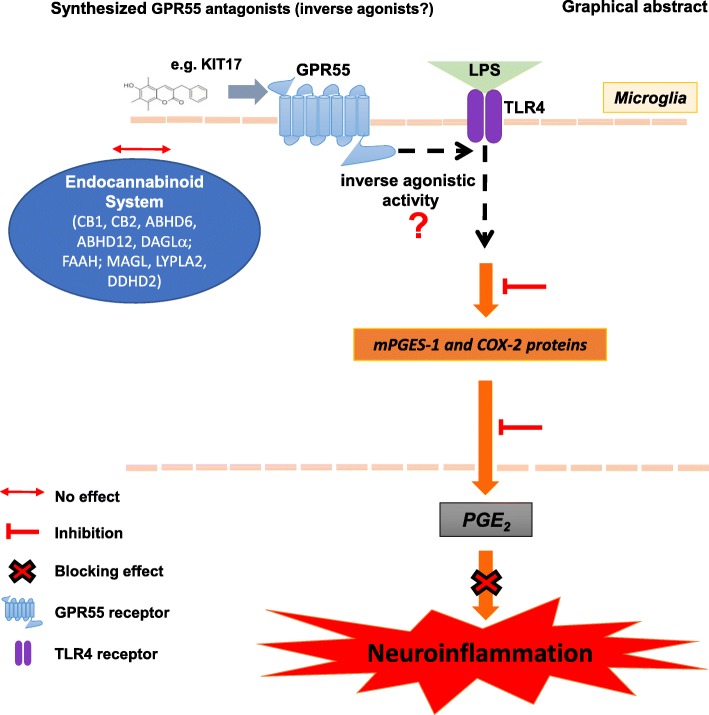
Graphical abstract showing the anti-neuroinflammatory effects of the KIT 17 in LPS-activated microglia. The anti-inflammatory effects of KIT 17 included the reduction of COX-2/mGES-1 levels as well as potent inhibition of PGE_2_ production

As illustrated in the graphical abstract (Fig. 8), we provide the evidence that KIT 17 and the other KIT coumarin derivates and GPR55 antagonists effectively block microglia activation in terms of attenuation of both PGE_2_ production and mPGES-1/COX-2 levels. Our data provide evidence that the anti-neuroinflammatory effects are mainly mediated by GPR55, most likely via an inverse agonistic activity. However, further experiments are necessary to better comprehend the mechanistic effects of KIT compounds and the involvement of GPR55 receptors. Our study suggests that GPR55 antagonists might be a new therapeutic option for the treatment of neuroinflammation-, neurodegeneration-, and neuroinflammation-related diseases.

## Additional files

Additional file 1: Expression of *GPR55* mRNA in primary rat microglia with or without LPS stimulation. Microglia were incubated with or without LPS (10 ng/mL) and after 4 h, *GPR55* mRNA expression was measured by qPCR. (PDF 7 kb)

Additional file 2: First screening of chemical-related synthesized compounds (KIT 1 - KIT 21) on LPS-induced PGE_2_ synthesis in microglia cells. (PDF 16 kb)

Additional file 3: Effects of KIT 17 on mRNA expression of *COX-1* in LPS-stimulated primary microglial cells. Cells were pre-treated with different concentrations of KIT 17 for 30 min before stimulating with LPS. After 4 h, *COX-1* was measured by qPCR (*n* = 5). *p < 0.01 with respect to LPS (one-way ANOVA followed by the Newman-Keuls post hoc test). (PDF 9 kb)

Additional file 4: Effects of the AM630 on PGE_2_ release in LPS-stimulated primary microglial cells. Microglia were pre-treated with AM630 (0.1–10 μM) for 30 min, afterwards cells were incubated with or without LPS (10 ng/mL) for the next 24 h. At the end of incubation, cell supernatants were collected and release of PGE_2_ was measured by enzyme immune assay (EIA). Values are presented as the mean ± SEM of at least 3 independent experiments. Statistical analyses were carried out by using one-way ANOVA and Newman-Keuls post hoc test with ***p < 0.001 compared to LPS group. (PDF 9 kb)

## Abbreviations

2-AG: 2-Arachidonoylglycerol
ABHD6 and ABHD12: α,β-Hydrolase domain-containing 6 and 12
ABPP: Activity-based protein profiling
AD: Alzheimer’s disease
ATP: Adenosine triphosphate
BCA: Bicinchoninic acid
CB: Cannabinoids
CNS: Central nervous system
COX: Cyclooxygenase
DAG: Diacylglycerol
DAGLα: Diacylglycerol lipase-α
DMEM: Dulbecco’s modified Eagle’s medium
DMSO: Dimethyl sulfoxide
EIA: Enzyme immunoassay
ERK: Extracellular signal-regulated kinase
FAAH: Fatty acid amide hydrolase
GPR: G-protein-coupled receptor
IBD: Inflammatory bowel disease
IFN-γ: Interferon gamma
IL: Interleukin
LPI: L-α-Lysophosphatidylinositol
LPS: Lipopolysaccharide
MAGL: Monoacylglycerol lipase
mPGES: Microsomal prostaglandin E synthase
MS: Multiple sclerosis
NK: Natural killer
OHSC: Organotypic hippocampal slice cultures
PBS: Phosphate-buffered saline
PD: Parkinson disease
PG: Prostaglandin
PVDF: Polyvinylidene fluoride
RhoA: Ras homolog gene family member A
ROCK: Rho-associated protein kinase
SDS-PAGE: Sodium dodecyl sulfate-polyacrylamide gel electrophoresis
TAMRA-FP: Tetramethylrodamine 5-carboxamdio fluorophosphonate
TLR: Toll-like receptor
TNBS: Trinitrobenzene sulfonic acid
TNF: Tumor necrosis factor
