# Supplementary figures and images for: Anti-neuroinflammatory effects of GPR55 antagonists in LPS-activated primary microglial cells
Source: J Neuroinflammation. 2018 Nov 19;15:322. doi: 10.1186/s12974-018-1362-7 (PMC6240959; doi:10.1186/s12974-018-1362-7)

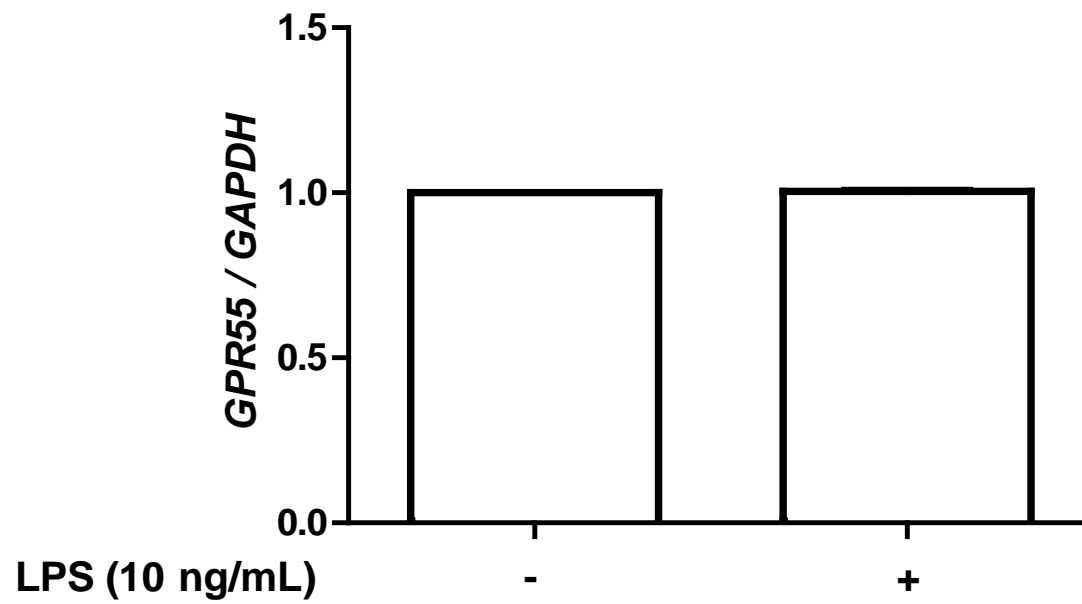

Supplement: Supplementary file 1 — Expression of GPR55 mRNA in primary rat microglia with or without LPS stimulation. Microglia were incubated with or without LPS (10 ng/mL) and after 4 h, GPR55 mRNA expression was measured by qPCR. (PDF 7 kb) [file 12974_2018_1362_MOESM1_ESM.pdf]

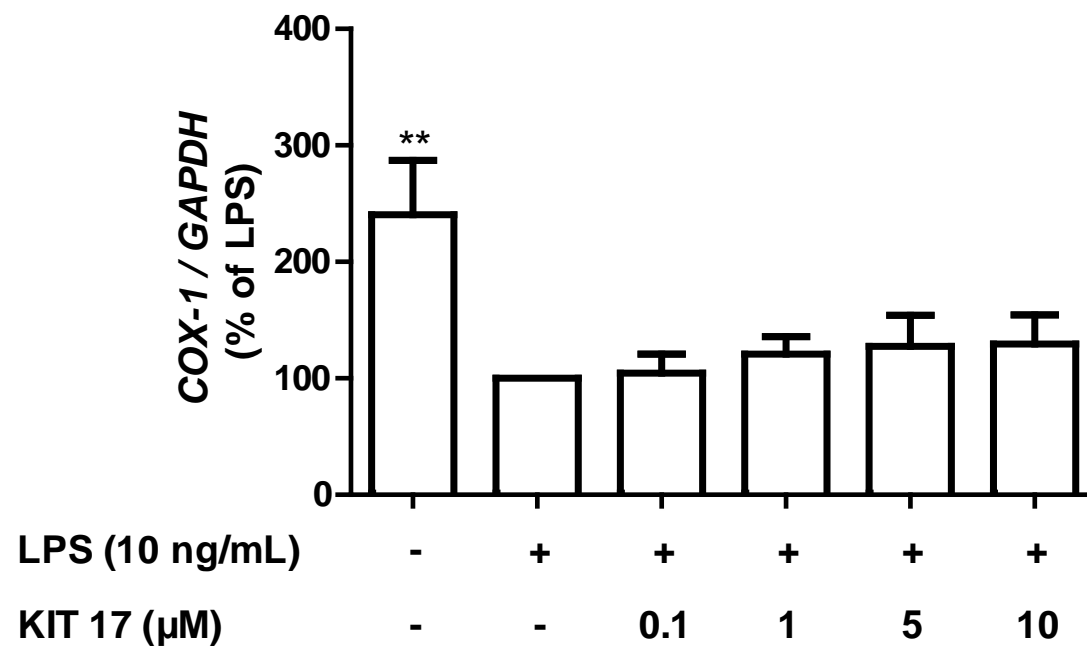

Supplement: Supplementary file 3 — Effects of KIT 17 on mRNA expression of COX-1 in LPS-stimulated primary microglial cells. Cells were pre-treated with different concentrations of KIT 17 for 30 min before stimulating with LPS. After 4 h, COX-1 was measured by qPCR (n = 5). *p < 0.01 with respect to LPS (one-way ANOVA followed by the Newman-Keuls post hoc test). (PDF 9 kb) [file 12974_2018_1362_MOESM3_ESM.pdf]

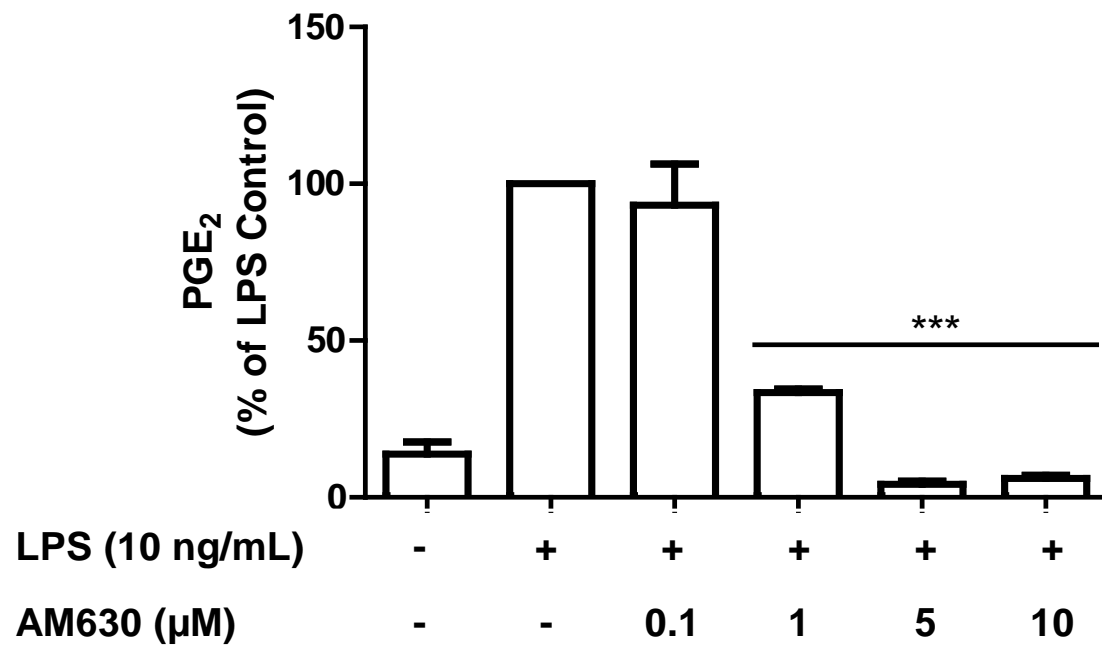

Supplement: Supplementary file 4 — Effects of the AM630 on PGE2 release in LPS-stimulated primary microglial cells. Microglia were pre-treated with AM630 (0.1–10 μM) for 30 min, afterwards cells were incubated with or without LPS (10 ng/mL) for the next 24 h. At the end of incubation, cell supernatants were collected and release of PGE2 was measured by enzyme immune assay (EIA). Values are presented as the mean ± SEM of at least 3 independent experiments. Statistical analyses were carried out by using one-way ANOVA and Newman-Keuls post hoc test with ***p < 0.001 compared to LPS group. (PDF 9 kb) [file 12974_2018_1362_MOESM4_ESM.pdf]
