# Supplementary material for: Anti-neuroinflammatory effects of GPR55 antagonists in LPS-activated primary microglial cells
Source: J Neuroinflammation. 2018 Nov 19;15:322. doi: 10.1186/s12974-018-1362-7 (PMC6240959; doi:10.1186/s12974-018-1362-7)
Supplement: Supplementary file 2 — First screening of chemical-related synthesized compounds (KIT 1 - KIT 21) on LPS-induced PGE2 synthesis in microglia cells. (PDF 16 kb) [file 12974_2018_1362_MOESM2_ESM.pdf]

Additional file 2: Screening of 21 chemical related GPR55 receptor antagonists (KIT 1 - KIT 21) on LPS-induced PGE_2 synthesis in microglial cells.

Compound	Corresponding number according Rempel et al., 2013	Prostaglandin E_2 Release Inhibition 100 µM
KIT 1	35	**
KIT 2	13	*
KIT 3	14	***
KIT 4	Unpublished	*
KIT 5	29	***
KIT 6	Unpublished	*
KIT 7	Unpublished	**
KIT 8	Unpublished	*
KIT 9	Unpublished	*
KIT 10	Unpublished	***
KIT 11	38	*
KIT 12	39	***
KIT 13	Unpublished	*
KIT 14	Unpublished	***
KIT 15	Unpublished	*
KIT 16	Unpublished	**
KIT 17	37	***
KIT 18	27	***
KIT 19	Unpublished	**
KIT 20	Unpublished	**
KIT 21	41	***

* 10-50% inhibition
** 50-90% inhibition
*** 90-100% inhibition
